# Biomimetic Artificial Joints Based on Multi-Material Pneumatic Actuators Developed for Soft Robotic Finger Application

**DOI:** 10.3390/mi12121593

**Published:** 2021-12-20

**Authors:** Shumi Zhao, Yisong Lei, Ziwen Wang, Jie Zhang, Jianxun Liu, Pengfei Zheng, Zidan Gong, Yue Sun

**Affiliations:** 1 Sino-German College of Intelligent Manufacturing, Shenzhen Technology University, Shenzhen 518118, China; 2070412003@email.szu.edu.cn (Y.L.); 2070412024@email.szu.edu.cn (Z.W.); 2110412004@stumail.sztu.edu.cn (J.Z.); liujianxun2019@email.szu.edu.cn (J.L.); 2070412009@email.szu.edu.cn (P.Z.); 2 Hefei Comprehensive National Science Center, Institute of Artificial Intelligence, Hefei 230026, China; zhaoshumi@iai.ustc.edu.cn; 3 School of Fashion Design & Engineering, Zhejiang Sci-Tech University, Hangzhou 310018, China; sunyue@zstu.edu.cn

**Keywords:** biomimetic artificial joints, multi-material actuator, pneumatic bellows, mathematical model, soft robotic finger

## Abstract

To precisely achieve a series of daily finger bending motions, a soft robotic finger corresponding to the anatomical range of each joint was designed in this study with multi-material pneumatic actuators. The actuator as a biomimetic artificial joint was developed on the basis of two composite materials of different shear modules, and the pneumatic bellows as expansion parts was restricted by frame that made from polydimethylsiloxane (PDMS). A simplified mathematical model was used for the bending mechanism description and provides guidance for the multi-material pneumatic actuator fabrication (e.g., stiffness and thickness) and structural design (e.g., cross length and chamber radius), as well as the control parameter optimization (e.g., the air pressure supply). An actuation pressure of over 70 kPa is required by the developed soft robotic finger to provide a full motion range (MCP = 36°, PIP = 114°, and DIP = 75°) for finger action mimicking. In conclusion, a multi-material pneumatic actuator was designed and developed for soft robotic finger application and theoretically and experimentally demonstrated its feasibility in finger action mimicking. This study explored the mechanical properties of the actuator and could provide evidence-based technical parameters for pneumatic robotic finger design and precise control of its dynamic air pressure dosages in mimicking actions. Thereby, the conclusion was supported by the results theoretically and experimentally, which also aligns with our aim to design and develop a multi-material pneumatic actuator as a biomimetic artificial joint for soft robotic finger application.

## 1. Introduction

Soft actuators have been widely applied in soft artificial muscles [1], soft gripper [2] and wearable rehabilitation [3] since they are compliant and intrinsically suited for contacting soft tissue [4]. The soft actuators commonly consist of internal channel(s) for pneumatic or hydraulic supply and reinforced with fiber to improve actuation [5]. However, when compared with pneumatic supply, soft actuators driven by hydraulic supply usually have a heavier structure with limitations in degrees of freedom and bending motion. Thereby, the soft pneumatic actuator is lightweight, flexible, and compatible for human–machine interactions [6], and the inner pneumatic chambers could offer a smooth and flexible bending motion that makes them the ideal components of soft robotic fingers [7,8]. The extensively used materials for soft actuators [9], such as electroactive polymers [10], shape memory polymers and alloys [11], and hyper-elastic elastomers [12], could provide flexibility and output force in the deformation. However, the inherent low stiffness in the soft actuator usually could not provide enough force output, which results in failures in object grasping [13]. Although actuators with high stiffness could bear a heavy load, they have some limitations in adapting to diverse shapes of objects during manipulating in practical applications [14]. Pressure-driven soft actuating materials rely on external pneumatic forces to spatially strain the actuator for working [15,16]. This actuation scheme requires a stiffness gradient or a special structure design to generate anisotropic motions and more sophisticated deformations [17,18]. Purely elastic materials do not dissipate energy when a load is applied and thus exhibit virtually no loss modulus [19]. By contrast, viscoelastic materials (such as polydimethylsiloxane [PDMS]) exhibit viscous and elastic properties [20,21]. Rehman et al. [22] used to develop a novel monolithic PDMS-based dual-channel bellows-structured pneumatic actuator that fabricated through a sacrificial molding technique which has also been adopted for the fabrication of microfluidic device in our previous study [23]. Additionally, the commercially available product PneuNets adopted molded elastomeric material and attach it to a stiffer material to make the multi-material actuator with an extensible top layer and an inextensible bottom layer [24]. Commonly, multi-material actuator composed of soft and stiffer materials to make good use of their characteristics, while the soft one generates deformation and the stiffer to restrict swelling effect and support bending. Therefore, a novel pneumatic actuator design based on multi-materials could improve its robustness and repeatability in performance.

With respect to the design methods, the soft actuator are usually measured through experiments or analyzed using mathematical and finite element model [25,26]. However, the finite element model is difficult to construct because of the highly nonlinear characteristics of the applied material and the complex coupling between the human fingers and the actuator [27]. Polygerinos et al. [28] recently presented a quasistatic model for a soft fiber-reinforced actuator to analyze the bending angle and the contacting force of object. Ding et al. [29] proposed a soft multi-material pneumatic actuator on the basis of principal strain field. Kumar et al. [30] designed a flexible bandage system on the basis of a polymeric stress memory actuator, and Ghate et al. [31] designed a bendable, soft, pneumatic silicone rubber actuator prototype and built a corresponding mathematical model to characterize its mechanical behavior. In addition, Taigo et al. [32] proposed a parametric model for extensible pneumatic actuator with bellows that have a long strain in one direction. Therefore, modeling is necessary in developing actuators because it could help generate geometrical design parameters and predict the bending angle and material selection in fabrication, thus reducing the cost and experimental exploration time [27,33,34]. However, different materials and structures need varying models, and pneumatic actuators with simple structure could easily bend into an arc, which is inconsistent with the curved contour of the finger [35,36,37].

Given the complexity of the finger motion, it is essential to consider the natural skeletal structure of human finger in order to achieve perfect compliance of soft robotic digit [38]. The human finger structure contains three interphalangeal joints, namely, distal (DIP), proximal (PIP), and metacarpal (MCP) joints [35,39]. Based on previous studies [8,40], the ranges of motions (ROMs) of finger joints among different individuals are approximately the same level, and the maximum flexion values for the MCP, PIP, and DIP joint ranges are approximately 95°, 110°, and 90°, respectively [41]. Thus, for the development of biomimetic artificial joints for a soft robotic digit, three sections of multi-material pneumatic actuators were essential, except the thumb with two joints. Furthermore, anthropomorphic robotic digit used multi-materials to place on each joint of the finger, matching the bending of the natural skeletal structure and its inherent similarity to the human finger. This can potentially be applied in a wide range of fields, from automatic finger operation to healthcare robot exploration.

The present study aims to design and develop multi-material pneumatic actuators as biomimetic artificial joints for soft robotic finger application. The multi-material pneumatic actuator mainly contains two parts (i.e., pneumatic bellows and PDMS frame constraint structure) and developed on the basis of two composite materials with slightly different shear modules. The bellows were partitioned into several compartments to achieve differential stretching when applying different pressures to each chamber to obtain different bending angles when the freedom of one is limited. The mechanical properties of the multi-material pneumatic actuators were analyzed using a simplified mathematical model and experimentally tested. A simplified mathematical model was adopted for the bending mechanism description; to provide guidance for actuator fabrication (e.g., stiffness and thickness), structural design (e.g., cross length and chamber radius), as well as the control parameter optimization (e.g., the air pressure supply); and to precisely achieve a series of daily finger bending motions mimicking the trajectory of human fingers.

## 2. Materials and Methods

### 2.1. Multi-Material Pneumatic Actuator Design and Fabrication

**Figure 1:**
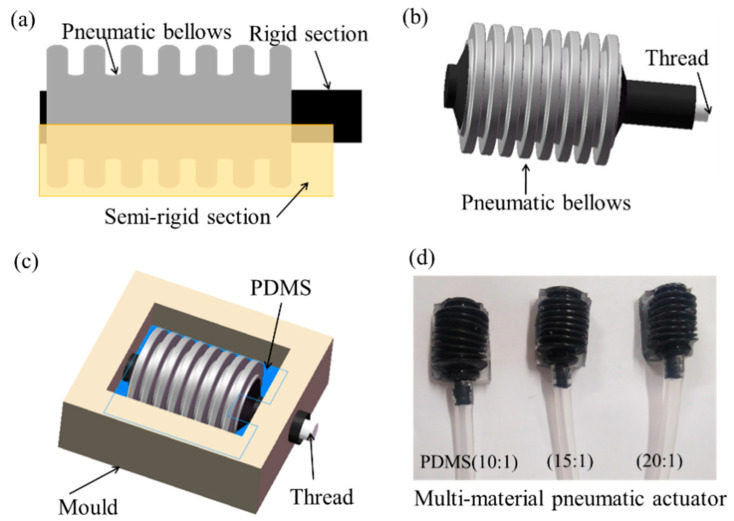
Multi-material pneumatic actuator design and manufacturing: (**a**) multi-material pneumatic actuator structure; (**b**) actuator reinforcement deployment; (**c**) actuator molding; (**d**) actuator with different stiffnesses.

The multi-material pneumatic actuator was engineered into three parts of different stiffness materials, i.e., pneumatic bellows, rigid section, and semi-rigid sections, as shown in Figure 1a. The designed pneumatic bellows with flexible chambers are made of silicone rubber material (SY-HR02, Shanghai Si Yi Intelligent Technology Co., Ltd., Shanghai, China), and located between two rigid sections in alternating order. The semi-rigid section was under the flexible chambers. This geometry allows the bending motions in one direction during air inflation and recovery during deflation. The hybrid architecture of the multi-material pneumatic actuator provides specific mechanical characteristics required by individual finger arthrosis to realize the desired bending motions.

The manufacturing process of the multi-material pneumatic actuator mainly includes three steps. The first step is reinforcement shaping of the adopted pneumatic bellows (Figure 1b). A thread was embedded into the bellow chamber center that helps keep the bellows in place. Afterwards, the pneumatic bellows were fixed and covered with fluidic PDMS (Dow Corning 184, Midland, MI, USA), as shown in Figure 1c. When compared with other materials, PDMS has a typically low surface interfacial free energy, good elastic characteristic as well as chemical inertness and durability, thus could be easily fabricated by molding [20,21,23]. The PDMS reagent contained a vinyl-terminated base and a curing agent (cross-linker agent) in two liquid component kits. The curing agent was added into the vinyl-terminated base and mixed together to make PDMS mixtures at the following weight ratios (vinyl-terminated base: curing agent) of 20:1, 15:1, and 10:1 respectively (the 10:1 ratio was suggested by the Sylgard 184 datasheet [42]). Trapped bubbles may form due to the different viscosities of the vinyl-terminated base (−5 × 10^−3^ m^2^ s^−1^) and the curing agent (1.1 × 10^–4^ m^2^ s^−1^) and need to be removed before molding and curing by placing in a low-vacuum chamber (−100 kPa) to degas for several minutes. The prepared PDMS mixtures were then injected into the mold for curing for 2 h at 70 °C. After that, the pneumatic bellows with PDMS substrate were removed from mold as well as the embedded thread. In the last step, an air tube was connected to the open side of the bellows and assembled a single multi-material pneumatic actuator as shown in Figure 1d.

### 2.2. Actuator Mathematical Model Design

**Figure 2:**
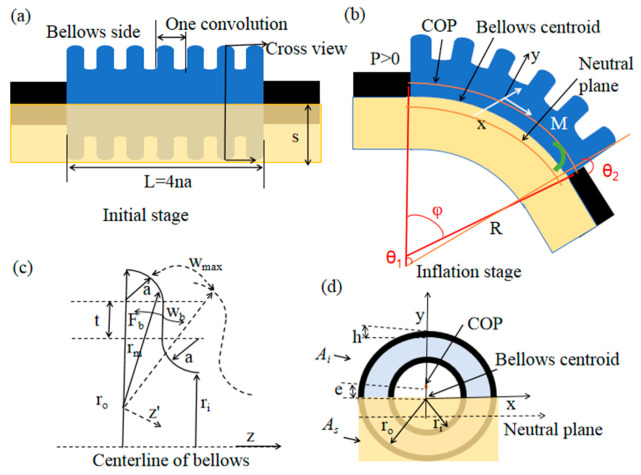
Bending analysis of multi-material pneumatic actuator: (**a**) initial stage; (**b**) subjected internal pressure to bend; (**c**) one convolution bending analysis; (**d**) actuator cross-section view.

The multi-material pneumatic actuator could be considered as a flexible semicircular bellow placed on a flat plate as presented in Figure 2a. When air pressure *P* was applied in the actuator with the free end closed, the actuator bended (Figure 2b) due to the differential expansion (e.g., one convolution expansion shown in Figure 2c) of the top bellows and bottom plate. *P* delivered a force *F* = *P* × *A_i_* to the actuator, where *A_i_* is the internal top bellow chamber’s cross-sectional area. Given the combined effect of PDMS frame constraint structure, the pressure center was slightly shifted from the bellow centroid by a small distance “*e*”, and the neutral planer was also shifted (Figure 2d).

The deflection of the multi-material pneumatic actuator was caused by the internal pressure action and the corresponding deformed geometry structure. Based on the bellows and beam theory [43,44], the vertical deflection *W* at the tip of the bellows and the angular deflection *θ*_1_ are given in terms of *M* as

(1) $$\theta_{1}=\frac{ML}{EI_{xa}}$$

where *M* indicates the moment acting at the free end, *I_xa_* is the area moment of inertia of the cross section of both bellows and the substrate, *E* is the Young’s modulus, and *L* is the length of the bellows.

The multi-material pneumatic actuators exhibited different stiffnesses, assuming they keep constant during working. The pressure force applied to the multi-material pneumatic actuator was separated into *F_b_* and *F_p_* for top and bottom bellows with PDMS, respectively. The total force was calculated as

(2) $$F=F_{b}+F_{p}=K_{b}w_{b}+K_{p}w_{p}$$

where $w_{b}\;$ and $w_{p}$ are the deflections, and *K_b_* and *K_p_* are the corresponding axial stiffness at the top bellow side and bottom flat side, respectively. When the bottom flat was assumed to be a whole block while ignored the embedded bottom bellows, then *K_p_ = (E*_2_ *× A_s_)/L*, where *A_s_* is the cross-section area of the substrate and *E*_2_ is the Young’s modulus of PDMS.

A simplified geometry for bending analysis in one bellow convolution is shown in Figure 2c. The stiffness of the top bellow expansion was defined as *K_b_ = B × I_xb_* in Hermann et al.’s research [45], where *B* is a constant which related to the geometry and the material properties of the bellow. *B* could be calculated as follows:

(3) $$\begin{array}{ll} B= & \frac{24E_{1}(4.602+6\times 10^{7}a^{3}-86.2r_{0})}{4n[6\pi a^{3}+24ta^{2}+t^{3}+3t^{2}a\pi (1+h^{2}/12a^{2})]}\\ I_{xb}= & \frac{\pi (r_{0}+r_{i})h^{3}}{12}=\frac{\pi r_{m}h^{3}}{6} \end{array}$$

where *a*, *t*, and *h* indicate the radius of corrugation, flank distance, and bellow thickness, respectively; *r*_0_ and *r_i_* are the outer and inner radius of the bellow; *r_m_* is the average radius of the bellow; *E*_1_ means the modulus of bellows; and *n* is the number of convolutions of bellows. Thus, the angular deflection *θ*_2_ generated by inner deformation was obtained as


(4) $$\theta_{2}=\frac{w_{b}-w_{p}}{r_{m}}$$

The total angular deflection of the multi-material pneumatic actuator in bending progress could be summarized by Equations (1) and (4) as


(5) $$\varphi =\theta_{1}+\theta_{2}$$

When the bellow expansion generated the moment *M*_exp_, the deflection force that varies around the circumference is multiplied by the corresponding levers and integrated around the circumference [44]. Thus, at any location of the bellows, an element force *dF* was equivalent to that exist at the average diameter. The moment *M*_exp_ is determined as

(6) $$M_{\exp}=\int dFr_{m}\sin\;\alpha =\frac{w_{\max}Bh^{3}r_{m}^{2}}{6}\int_{0}^{\pi }\sin^{2}\;\alpha d\alpha =\frac{w_{\max}B\pi h^{3}r_{m}^{2}}{12}$$

where ω_max_ is the maximum defection at the top of bellows: ω_max_ *= rm* *θ*_2_. The total moment of the multi-material pneumatic actuator due to *P* could be obtained as


(7) $$M=F\times e+M_{\exp}$$

When the multi-material pneumatic actuator was inflated with air pressure, *φ* became larger due to actuator bending. Thus, the model–experiment method for the actuator could be used to predict the bending angle of the multi-material pneumatic actuator from a quantitative or qualitative perspective.

### 2.3. Soft Robotic Finger Design and Fabrication

**Figure 3:**
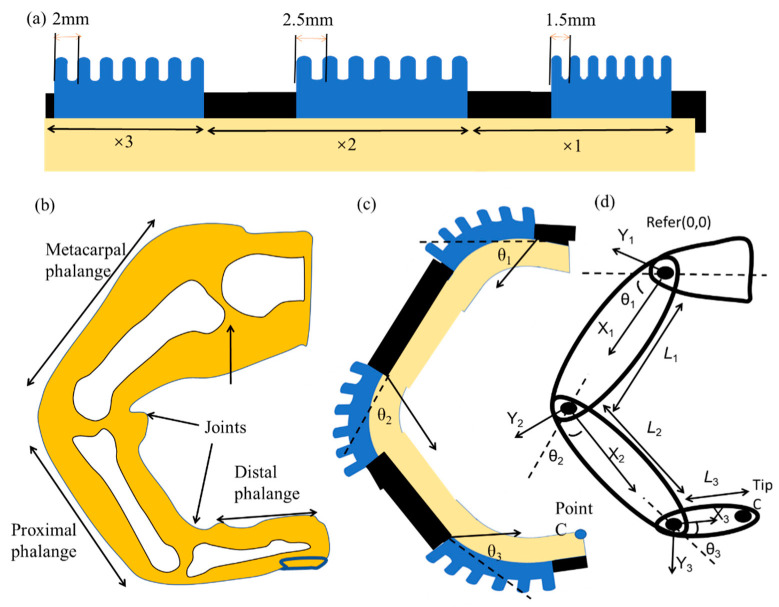
Finger exoskeleton robots: (**a**) Schematic of a single digit with three joints; (**b**) bone structure of a human finger; (**c**,**d**) Kinematic model of the robotic digit.

To mimic finger action, it is essential to study the natural skeletal structure of the human finger [8,46]. The finger mainly contains three interphalangeal joints, namely, distal phalange (DIP), proximal phalange (PIP), and metacarpal phalange (MCP) joints [35,39]. According to previous studies [40], the ROMs of finger joints among different individuals are approximately the same. Thus, three sections of pneumatic bellows were necessary for the soft robotic digit to place on each joint of finger matching the natural skeletal structure. Figure 3a shows the robotic digit and its components (different types of multi-material pneumatic actuators with different sizes). Refer finger bending stage Figure 3b, a simple kinematic model of the robotic digit was depicted in Figure 3c,d from a kinematic perspective.

**Figure 4:**
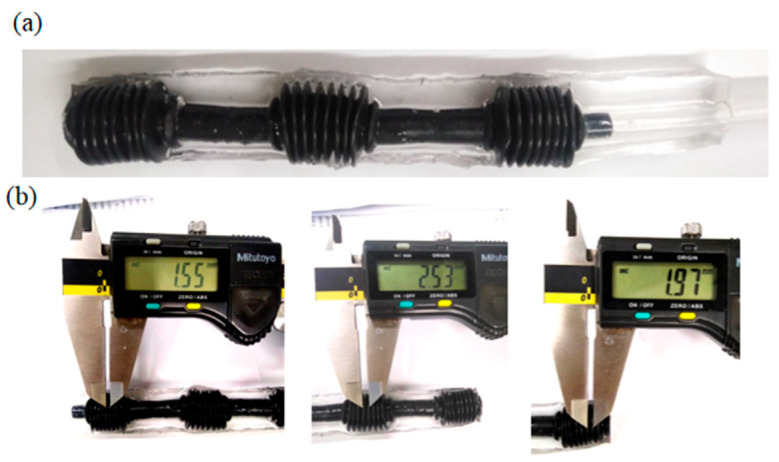
Soft robotic finger: (**a**) a fabricated soft robotic finger with three multi-material pneumatic actuators; (**b**) different size of each pneumatic actuator.

The multi-material pneumatic actuators were assembled for the robotic finger, and the manufacturing process followed three steps. Firstly, for reinforcement shaping, a thread was embedded into the bellow center to keep the bellows on the same axis. Secondly, after the pneumatic bellows were fixed, they were covered with fluidic PDMS with the curing parameter setting similar to that of single-actuator fabrication. Lastly, when PDMS was cured, the external mold was opened to gently remove the three-part pneumatic bellows and connect an air tube to the open side of the bellows as shown in Figure 4. Thus, a soft robotic finger was fabricated based on multi-material pneumatic actuators with special dimension, which could bend along the PDMS substrate downwards when air pressure is inflated.

## 3. Result

### 3.1. Actuator Model for Material and Structural Parameter Analysis

Finite element methods are commonly adopted to simulate the pneumatic bellow actuator behavior for working mechanism analysis [47,48,49]. However, these methods require precise object description and complex computation [50,51]. In the current study, from the optimization perspective of the materials and structure, the simplified static mathematical model for multi-material pneumatic actuator analysis only involved a small set of calculations on the basis of some assumptions. As mentioned in Section 2.2, the bending angle is relative to the internal pressure of chamber in free end motions, which could be determined using Equation (5) as:


(8) φ=f(P)

Table 1 lists the values used in the model for Equation (10) calculation. The mathematical model showed that a multi-material pneumatic actuator could work under low pressure due to the rubber material. The model demonstrated good agreement with the experiment results thereby could be adopted for robotic finger unit design.

**Figure 5:**
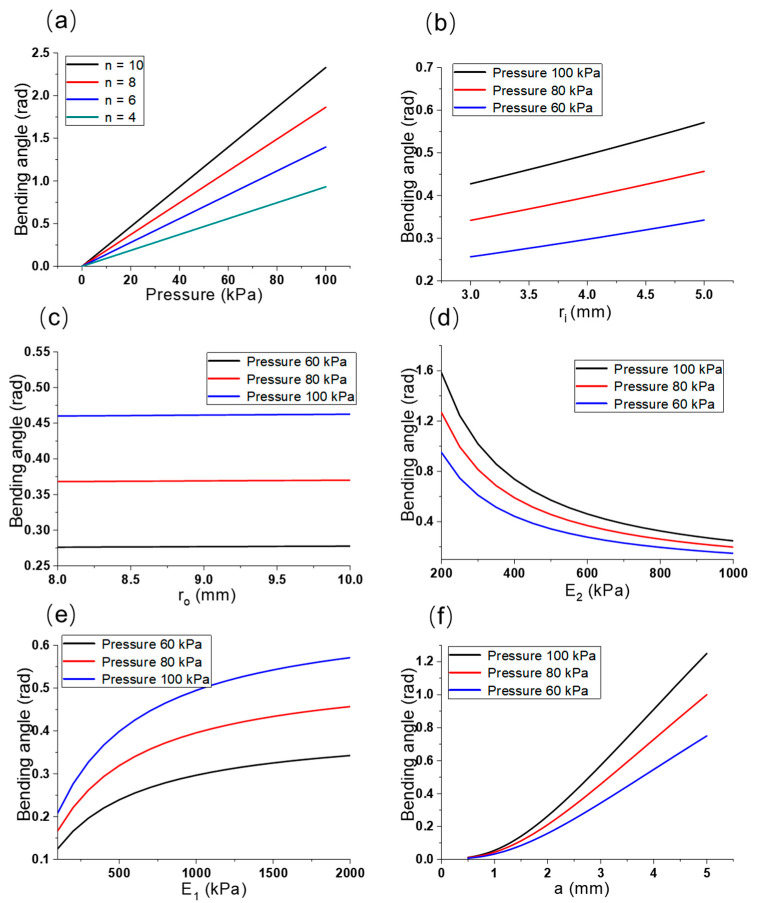
Bending angle correlation simulation towards: (**a**) bellow number, (**b**) small chamber radius, (**c**) large chamber radius, (**d**) substrate stiffness, (**e**) bellow stiffness, and (**f**) cross length of chamber.

Figure 5a–f present the interactions among the vital parameters of the multi-material pneumatic actuator revealed by MATLAB simulation (MATLAB, 2019b, MathWorks, MA, USA). Bending angle of the actuator increased along with the increased pneumatic pressure inside (Pa) as illustrated in Figure 5a. Under different pressure conditions (e.g., *P* = 100, 80, and 60 kPa), the bending angle of the bellows increased obviously with the increase in small chamber radius; conversely, the bending angle of the bellows increased slowly along with the increase in big chamber radius, as shown in Figure 5b,c. Moreover, Figure 5d,e present that when the stiffness of substrate PDMS increased, the bending angle of the actuator decreased, and when the stiffness of the top bellows increased, the bending angle of the actuator increased. Therefore, the material properties could affect the bending angle of actuators. In addition, the bending angle of the bellows increased along with the increase in the cross length of the chambers, as shown in Figure 5f. Thus, the effects of multiple factors (e.g., cross length of bellows, chamber radius, and stiffness) on multi-material pneumatic actuators could be quantitatively and qualitatively analyzed in accordance with the simulated results.

Material parameters, such as PDMS substrate with different stiffnesses and chambers of bellows in various geometries, could be utilized to tailor the bend angle of the pneumatic bellow and design the robotic digit in accordance with the requirements on specific finger applications. This study found that the key parameters (e.g., radius and structure of the bellow chambers, bellow stiffness, and PDMS substrate) and the pressure supply directly affect the bending angle of the pneumatic bellows.

### 3.2. Single Actuator Motion Range Analysis

**Figure 6:**
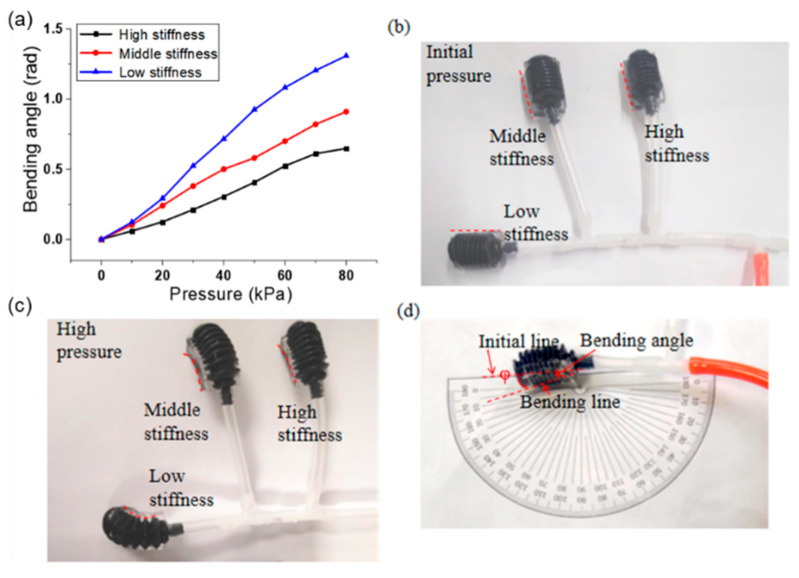
Bending angle test of multi-material pneumatic actuator: (**a**) bending result of actuator with various PDMS stiffnesses under different pressures; (**b**–**d**) actuator test examples.

Following the aforementioned processes Figure 1b–d there fabricated the single actuator with two different materials where the PDMS parts considered as the frame constraint structure for fixing the bottom side of the pneumatic bellow and limiting forward expansion. PDMS with different stiffnesses was used to explore its effect on bending performance of the pneumatic bellow. The PDMS mixtures under different weight content ratios upon mixing the vinyl-terminated base and the curing agent exhibited different stiffnesses, and previous research offered a range of reported PDMS Young’s modulus (280–750 kPa) [53,54]. The stiffness of the frame constraint structure increased along with weight content ratio of the PDMS mixtures. Thus, the bending angle of the multi-material pneumatic actuator differed under the same pressure as shown in Figure 6a. Three kinds of PDMS (i.e., 20:1, 15:1, and 10:1) that fabricated the multi-material pneumatic actuator were simultaneously tested for bending angles by using a tee connector under the same pressure (0–58.6 kPa), as shown in Figure 6b–d illustrates that the flexion angles were measured and calculated under a special given pressure.

Figure 6a shows the bending angles of the developed actuator increased with PDMS stiffness decrease under the pressure less than 80 kPa. However, the decreased PDMS stiffness (20:1) in the actuator bottom generated large deformation under high pressure (i.e., when the pressure was higher than 60 kPa, the length of multi-material pneumatic actuator in the center axis direction increased). Meanwhile, the increased PMDS stiffness (10:1) generated the same bending angle but needed higher pressure. A rigid PDMS was not suitable for the frame constraint structure of the multi-material pneumatic actuator. Therefore, PDMS fabrication technology with a weight content ratio of 15:1 was selected in this study for the fabrication of multi-material pneumatic actuators.

**Figure 7:**
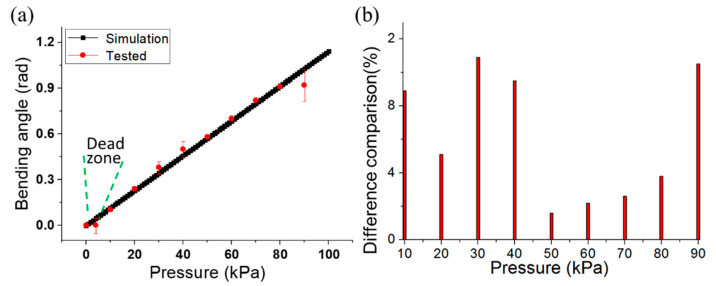
Actuator simulation and experimental result comparison analysis: (**a**) the simulated and experimental bending angle comparison; (**b**) difference analysis between simulation and experimental result.

Figure 7a presents the comparison between the proposed model (Equation (10)) and the experimental results. There is a dead zone caused by the material deformation properties at the first stage. Additionally, the relative error variations of the flexion angles were calculated to explore the consistency of bellows under repeated pressure test as shown in Figure 7b. The relative error can be determined as e=Δθ/*θ*_0_ at a certain supplied pressure, where ∆*θ* indicates the maximum deviation of the flexion angle and *θ*_0_ represents the average flexion angle. The difference between the simulation and the experimental results possibly due to the elimination of the PDMS base weight, and the effect of the dynamic stiffness of the actuators during the experiments. However, when the pressure was higher than 80 kPa, the deformation of the bottom bellows embedded in the substrate could not be ignored.

By comparing the experimental and simulation results, the PDMS (15:1) for multi-material pneumatic actuator showed an acceptable performance towards the bending angle and lower required pneumatic pressure. The results demonstrated that when the bellow stiffness decreased there produce forward expansion and the required bending angle design could not be realized; when the stiffness increased, the bellow needed higher pressure to produce bending. Additionally, and simulated and experimental results revealed that when the current stiffness of the frame constraint structure increased, the bending angle would decrease. Thus, the target bending shape required in multi-material pneumatic actuators could be achieved by fine tuning the PDMS stiffness and the supply pneumatic pressure. Moreover, when compared with aforementioned existing actuators [22,28,29], our multi-material pneumatic actuator has better integration, simpler fabrication technique and more ergonomic considerations in application. Additionally, when compared with the commercially available bellow actuator, PneuNets (Soft robotics toolkit, MA, USA), which adopted molded elastomeric material and stiffer material, our actuator is more stable and easier to be assembled as artificial joints in a soft robotic finger application.

### 3.3. Finger Motion Range Analysis

**Figure 8:**
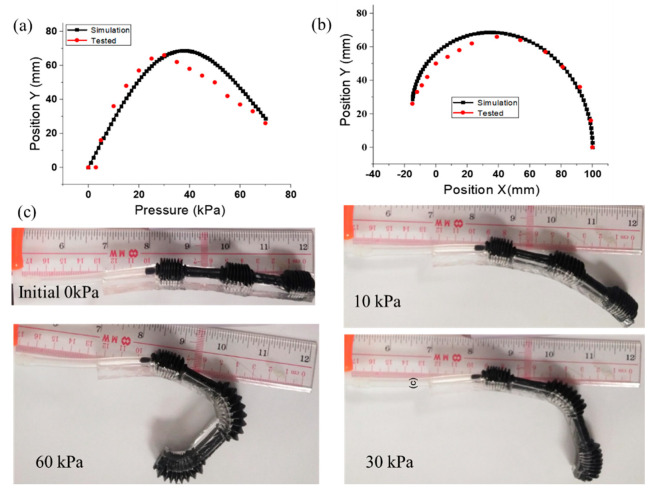
The experimental and kinematic model simulation comparison analysis: (**a**) in y direction; (**b**) finger trajectory; (**c**) soft robotic digit mimicking finger action (four types, from extension to flexion) under different pressure.

The MCP, PIP, and DIP joints were fixed with different chamber-structure bellow actuators with chamber cross widths of 1.5, 2.5, and 2 mm, respectively. The ROMs of the different bellows were highly dependent on the pressure supply when bellows structure was fixed. References were set up based on the finger frames and assigned to this model to depict the bending actions of joint points and the tip point C (Figure 3). The position variation of the tip point in the bending direction could be described using its components as follows:

(9) $$x_{c}=L_{1}\cos\phi_{1}+L_{2}\cos(\phi_{1}+\phi_{2})+L_{3}\cos(\phi_{1}+\phi_{2}+\phi_{3})\;$$

(10) $$y_{c}=L_{1}\sin\phi_{1}+L_{2}\sin(\phi_{1}+\phi_{2})+L_{3}\sin(\phi_{1}+\phi_{2}+\phi_{3})\;$$

where *φ*_1_, *φ*_2_, and *φ*_3_ are the first, second, and third actuator joint bending angles, and *L*_1_, *L*_2_, and *L*_3_ are the lengths of DIP, PIP, and MCP, respectively. The trajectory of the digit tip and the range motion of each joint were simulated and measured in the robotic digit experiments (Figure 8), in which different points were tested under various constant pressure supplies.

Experiments on soft robotic digit mimicking the finger action (four types, from extension to flexion) were conducted as presented in Figure 8a–c, which also presents a comparison of the robotic digit tip trajectory in the experiment and the kinematic model simulation. The two trajectory path plots behaved in the same trend and exhibited a similar inflection point. Although the tip trajectory had a small deviation under high air pressure (more than 40 kPa), the main reason is that the expansion of the bottom bellows cannot be ignored. Therefore, based on the established kinematic model, the finger actuators can be customized for different applications by different pressure supply.

The measured motion range of the soft robotic digit not only satisfied the finger bending range but also was close to the reported anatomical motion range and the simulated results in Figure 8c. The slight variations between the experimental and simulation results could be attributed to the friction between the fabricated robotic digit and the flat supporting surface during flexion and extension. Therefore, an actuation pressure of over 70 kPa is required to provide a full motion range of soft robotic finger. Moreover, the achieved motion ranges (MCP = 36°, PIP = 114°, and DIP = 75°) are consistent with the functional ROM of the human finger for performing daily activities [55,56].

**Figure 9:**
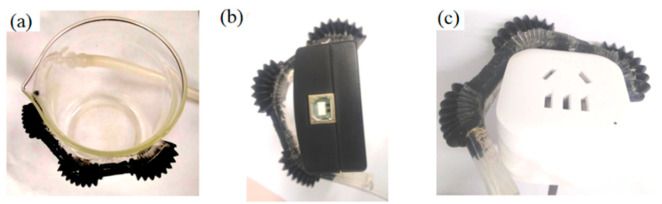
Soft robotic digit grasp 3 types of objects: (**a**) beaker, (**b**) USB box, and (**c**) socket.

The multi-material pneumatic actuators based soft robotic finger match with the bending changes in the anatomy of the human finger structure. In the range of soft robotic digit motion experiments, the MCP joint has a small motion range and the DIP and PIP joints have a wider motion range when it grasped many different objects with various shapes (such as beaker, USB box, socket, etc.) as shown in Figure 9a–c, which is matched with the theoretical design. These results showed that the developed soft robotic digit has the potential to assist gripping movements.

The bending experiments on the basic movement of the soft robotic finger and the experiments of gripping many different objects demonstrated that soft robotic digit mimicking finger action could be achieved by using the multi-material pneumatic actuator. Thus, the extension and flexion mechanism of the soft robotic digit could be easily controlled by pneumatic pressure. The object grasp experiment demonstrated that the developed soft robotic finger is not only be able to mimic finger flexion and extension but also grasp some daily objects. The primary focus of this work was the development and optimization of the multi-material pneumatic actuator for the soft robotic finger motion mimicking. In the future, we will evaluate the soft robotic finger for basic gripping movement assisting patients who have difficulty with finger movements.

## 4. Conclusions

This study proposed a multi-material pneumatic actuator based on simplified mathematical model for soft robotic finger application and theoretically and experimentally demonstrate its feasibility in mimicking human finger motions. The actuator was designed to satisfy the anatomical range of finger motion for each joint, and the simplified mathematical model was used to analyze the robotic finger motion and therefore predict the design and fabrication parameters, such as the pressure supply and the material requirements. The multi-material pneumatic actuator as a biomimetic artificial joint was developed on the basis of two composite materials with different shear modules, meanwhile pneumatic bellows as expansion parts was restricted by frame which was made from PDMS. The three-step fabrication progress method provided an easy and fast technique for casting such multi-material models. The geometry of each robotic finger with three multi-material pneumatic actuators allowed forward and backward bending motions during pneumatic pressure inflation and deflation, respectively. The effectiveness of the proposed soft robotic finger was validated by the experimental results which demonstrated the bending characteristics of the multi-material pneumatic actuators under different pneumatic pressures. Theoretical models and experimental analysis were both conducted to investigate the mechanical properties of the developed actuator and could provide evidence-based technical parameters for pneumatic robotic finger design and the precise control of its dynamic air pressure dosages in mimicking actions. In the future, the adopted multi-material pneumatic actuator could be optimized to explore the interaction (motion and force) between human finger and soft robotic digit for assistive applications.

## Figures and Tables

**Table 1:** Simulation parameters of multi-material pneumatic actuator.

Param.	Definition	Value
*a*	Bellow arch	0.3 × 10^−3^ m
*h*	Thickness of the rubber	0.4 × 10^−3^ m
*t*	Flank distance	1 × 10^−3^ m
*r_i_*	Representative radius of small chambers	5 × 10^−3^ m
*r*_0_	Representative radius of large chambers	8 × 10^−3^ m
*s*	Thickness of the substrate	8 × 10^−3^ m
*n*	Number of bellows	4
*E*_1_	Young’s modulus of silicone rubber	2 × 10^6^ Pa [52]
*E*_2_	Different Young’s modulus of PDMS	(280–750 kPa) [53,54]
*P*	Supply pressure	100 kPa

## Data Availability

The data presented in this study are available on request from the corresponding author.
